# Navigating the Philippine mental health system for the nation's youth: challenges and opportunities

**DOI:** 10.1192/bji.2024.5

**Published:** 2024-08

**Authors:** Rowalt Alibudbud

**Affiliations:** Department of Sociology and Behavioral Science, De La Salle University, Manila, Philippines. Email: rowalt.alibudbud@dlsu.edu.ph

**Keywords:** Mental health system, mental health services, Philippines, youth, Mental Health Act

## Abstract

The challenges besetting the Philippine mental health system demand multifaceted, strategic responses to ensure the holistic well-being of Filipino youth. Through the integration of mental health into primary care, augmentation of the professional workforce, bolstering information infrastructure, reforming medication accessibility, augmenting budgetary allocations and invigorating governance, the Philippines can pave the way for an inclusive mental health system that adequately addresses the exigencies of its younger demographic. In doing so, the nation can make substantial strides towards alleviating the negative impacts of adverse social conditions, such as the COVID-19 pandemic, on the mental well-being of its youth.

The repercussions of adverse social conditions, including social unrest and the global COVID-19 pandemic, have engendered lasting implications for the mental well-being of youth.^1^ Notably, the Philippines, since grappling with one of the world's longest and strictest COVID-19-related lockdowns, has witnessed an exacerbation of pre-existing determinants of compromised mental health, exemplified by widespread poverty.^2,3^ Against this backdrop, it becomes imperative to proactively address the mental health adversities borne by about 20 million young Filipinos amid these adverse social circumstances.^4^ Early into the COVID-19 pandemic, evidence suggested that a notable 16.9 and 28.8% of the Philippine population grappled with moderate to severe depressive and anxiety symptoms.^5^ Notably, the vulnerability of young Filipinos to such distressing mental health conditions was particularly conspicuous.^5^ Among students, stress during the pandemic and the transition to online learning manifested in sleep disturbances and challenges with academic, social and recreational activities.^6^ As the pandemic's trajectory evolved, alarming statistics indicated that the number of young Filipinos who had thought of suicide more than doubled during the pandemic compared with pre-pandemic rates.^4,7^ Therefore, there is an imperative to delve into the challenges and opportunities faced by Filipino youth as they navigate the intricacies of the country's mental health system.

Although previous reviews, such as that conducted by Lally et al in 2019,^8^ have adeptly underscored the inherent challenges encountered by the Philippine mental health system, there is a notable dearth of analyses specifically accentuating the nuances of mental health services for youth. Moreover, although Lally et al provided a comprehensive examination of the Philippine mental health system in the immediate aftermath of the implementation of the Philippine Mental Health Act in 2019,^8,9^ there is a pressing need for an updated assessment, capturing the achievements and challenges encountered by the country's mental health system in the post-enactment period.

Addressing these imperatives, this paper elucidates the contemporary challenges and opportunities within the national mental health system, with a focus on the mental health needs of Filipino youth. Drawing from the World Health Organization framework for the building blocks of health systems,^10^ this paper situates the mental health system of the Philippines within several components: service delivery, workforce, information systems, accessibility, financing and governance.

## Mental health service delivery

The mental health services of the Philippines have traditionally been delivered by specialists in psychiatric hospitals, supplemented by out-patient services in general medical facilities.^11^ This structure might stem from the dearth of integration between primary healthcare and mental healthcare services.^8^ Notwithstanding, efforts have been made to rectify this disparity by integrating mental healthcare into primary care, a vision elucidated by the Mental Health Act.^9^ As of 2020, the World Health Organization (WHO) reported that 1134 local government units had at least one staff member trained in the Mental Health Gap Action Programme (mhGAP).^12^ Despite this progress, the WHO also highlighted a deficiency in providing training to primary healthcare physicians to recognise early signs of mental disorders.^12^

Furthermore, the advent of telemedicine during the COVID-19 crisis has opened new avenues for mental health service delivery.^11^ For instance, a Philippines-based university has introduced a mobile application to offer online counselling services.^6^ Complementing this initiative, the university has implemented online mental health surveys to assess the requirements of its stakeholders.^6^ Additionally, the institution has taken strides to enhance mental health promotion by organising webinars focused on promoting self-care and well-being.^6^ Therefore, scaling up training for primary care workers is indispensable to enhance this integration. Achieving this entails implementing a comprehensive ‘training of trainers’ initiative and fostering partnerships with established training institutions, such as hospitals offering psychiatric training. Recognising young people's significant time in educational institutions, seamless incorporation of mental health awareness and support services, including training in psychological first aid, within school settings can also be supported.

## Mental healthcare workforce

The Philippines confronts a glaring inadequacy of mental health professionals, manifesting in a substantial shortage of psychiatrists and psychologists compared with global benchmarks. In 2022, it was reported that there were only about 1600 registered psychologists and 500 psychiatrists for the Philippines’ more than 110 million population.^13^ This concerning situation is compounded by a shortage of other mental health professionals, as highlighted by the WHO in 2020, which underscored the limited presence of approximately 500 psychiatric nurses and 1200 social workers within the country.^12^ Notably, the shortage is even more pronounced for specialists catering to the younger population, with only about 60 child psychiatrists in the country.^14^ Mitigating these inadequacies necessitates incentivising the training of mental health professionals, possibly through scholarship programmes targeting aspiring practitioners seeking specialised training, such as psychiatric residency and psychology degrees. Alongside this, fostering the expansion of existing training programmes becomes pivotal in accommodating an anticipated upsurge in trainees.

## Mental health information system

Although the development of a comprehensive mental health information system in the Philippines remains a work in progress, nascent efforts have given rise to prototypes, such as the National Suicide Registry and the Mental Health Information System.^11^ Nonetheless, the Strategic Plan for Mental Health includes pivotal advancements in information technology. The data will be efficiently sorted by a digital hub, slated for placement at the National Center for Mental Health.^12^ At present, several mental health indicators, such as depression, psychosis, dementia, substance misuse and self-harm, are integrated into the existing health information system.^12^ Charting a path forward, bolstering these information systems to better cater to the youth demographic involves their seamless integration into primary care systems and the private mental healthcare and paediatric sectors.

## Mental healthcare accessibility

Challenges in accessing essential medications remain a concern in the Philippines. Despite recent governmental initiatives to enhance the availability of select psychotropics (i.e. the Medicine Program for Mental Health), existing regulations pose obstacles to efficient service delivery and medication accessibility.^11^ Regulatory hurdles, exemplified by the Dangerous Drugs Act of 2002, which mandates the exclusive issuance of prescription forms by the Department of Health for certain psychotropic medications (e.g. methylphenidate),^15^ present impediments to streamlined treatment in cases such as attention-deficit hyperactivity disorder.

Furthermore, although the WHO acknowledges the ready availability of essential antipsychotic, antidepressant, anxiolytic, mood-stabilising and anti-epileptic medications at specialist mental health facilities in the Philippines, deficiencies in medicine stocks may persist in community health centers.^12^ This is attributed to limitations in the government's information technology capacity and manual reporting in rural health units.^12^ Addressing this situation requires repealing counterproductive policies and establishing frameworks that facilitate efficient treatment and medication provision, especially for young Filipinos with mental disorders. Additionally, legislative efforts to reduce taxes and tariffs on psychotropics could enhance medication accessibility.

## Mental health financing

Historically, mental health funding in the Philippines has been meagre, underscored by a mere 2.65% allocation of domestic government health expenditure to mental health in 2017.^11^ Notably, the annual per capita government expenditure on mental health of US$0.47 lags behind the global median of US$7.49.^11^ More recently, a 2021 report has observed only marginal enhancements in mental health financing over the years, with a mere 5% of the total healthcare expenditure being channelled into mental health initiatives.^16,17^ Nevertheless, the year 2023 marks a pivotal moment, witnessing a substantial increase in central government funding from 57 million PHP (approximately US$1 million ) to 1 billion PHP (around US$20 million).^17,18^ Recognising the pivotal role of financial resources in enhancing mental health services and treatment accessibility, the augmentation and sustainability of mental healthcare investment is imperative. Diversification of funding sources, notably through leveraging health-related taxes such as those on tobacco and alcohol products, can potentially bolster the financial underpinning of mental health initiatives. This potential influx of additional funds could be allocated strategically to strengthen the growth of school-based mental health programmes and out-patient treatment packages.^18^

## Governance of mental healthcare

Governance of mental health services in the Philippines is entrusted to the Philippine Mental Health Council, instituted in 2018 under the Mental Health Act.^19^ Tasked with policy formulation, coordination and advisory responsibilities, this governmental body shoulders the responsibility of overseeing the rational, integrated and responsive delivery of mental health services to Filipinos.^19^ To optimise its efficacy, the council must proactively elevate its visibility and advocacy efforts. A judicious approach to this endeavour involves strategically disseminating innovative mental health programmes and initiatives through traditional and social media platforms.

## Conclusion

Overall, various challenges confronting the mental health system of the Philippines necessitate a comprehensive and strategic approach to safeguard the welfare of Filipino youth. This imperative underscores the significance of multifaceted interventions aimed at integrating mental health services within primary care frameworks, strengthening the professional workforce dedicated to mental health, enhancing the existing information infrastructure, effecting reforms in the accessibility of psychotropic medications, supplementing the fiscal allocations for mental health initiatives and enhancing the efficacy of the Philippine Mental Health Council. By diligently addressing these areas, the Philippines can establish a mental health system that aptly caters to the pressing needs of its increasing youth population. In this concerted endeavour, the nation stands poised to make notable advancements in mitigating the repercussions of adverse socio-environmental circumstances on the mental well-being of its youth.

## Data Availability

Data availability is not applicable to this article as no new data were created or analysed in this study.
